# Supplementary material for: Standardized on-road tests assessing fitness-to-drive in people with cognitive impairments: A systematic review
Source: PLoS One. 2020 May 18;15(5):e0233125. doi: 10.1371/journal.pone.0233125 (PMC7233547; doi:10.1371/journal.pone.0233125)
Supplement: S1 Protocol — (PDF) [file pone.0233125.s001.pdf]

National Institute for Health Research

UNIVERSITY of York
Centre for Reviews and Dissemination

Systematic review

1. * Review title.

Give the working title of the review, for example the one used for obtaining funding. Ideally the title should state succinctly the interventions or exposures being reviewed and the associated health or social problems. Where appropriate, the title should use the PI(E)COS structure to contain information on the Participants, Intervention (or Exposure) and Comparison groups, the Outcomes to be measured and Study designs to be included.

Standardized on-road instruments assessing fitness-to-drive in people with cognitive deficits: a systematic review

2. Original language title.

For reviews in languages other than English, this field should be used to enter the title in the language of the review. This will be displayed together with the English language title.

Outils de mesure standardisés évaluant l'aptitude à la conduite auprès de personnes présentant des troubles cognitifs: une revue systématique

3. * Anticipated or actual start date.

Give the date when the systematic review commenced, or is expected to commence.

15/06/2018

4. * Anticipated completion date.

Give the date by which the review is expected to be completed.

01/09/2019

5. * Stage of review at time of this submission.

Indicate the stage of progress of the review by ticking the relevant Started and Completed boxes. Additional information may be added in the free text box provided.
Please note: Reviews that have progressed beyond the point of completing data extraction at the time of initial registration are not eligible for inclusion in PROSPERO. Should evidence of incorrect status and/or completion date being supplied at the time of submission come to light, the content of the PROSPERO record will be removed leaving only the title and named contact details and a statement that inaccuracies in the stage of the review date had been identified.
This field should be updated when any amendments are made to a published record and on completion and publication of the review. If this field was pre-populated from the initial screening questions then you are not able to edit it until the record is published.

The review has not yet started: No

Review stage	Started	Completed
Preliminary searches	Yes	Yes
Piloting of the study selection process	Yes	Yes
Formal screening of search results against eligibility criteria	Yes	Yes
Data extraction	Yes	Yes
Risk of bias (quality) assessment	Yes	Yes
Data analysis	Yes	Yes

Provide any other relevant information about the stage of the review here (e.g. Funded proposal, protocol not yet finalised).

The review has been completed but the articles will be written following the master's thesis defense (end of June 2019).

The review has been completed but the articles will be written following the master's thesis defense (end of June 2019).

6. * Named contact.

The named contact acts as the guarantor for the accuracy of the information presented in the register record.

David Bellagamba

Email salutation (e.g. "Dr Smith" or "Joanne") for correspondence:

David

7. * Named contact email.

Give the electronic mail address of the named contact.

bellagamba.david@gmail.com

8. Named contact address

Give the full postal address for the named contact.

Rue des Cliniques 15, CH-1700 Fribourg, Switzerland

9. Named contact phone number.

Give the telephone number for the named contact, including international dialling code.

+41 79 221 68 53 or +41 78 845 34 59

10. * Organisational affiliation of the review.

Full title of the organisational affiliations for this review and website address if available. This field may be completed as 'None' if the review is not affiliated to any organisation.

School of Health Sciences, University of Applied Sciences and Arts Western Switzerland

Organisation web address:

https://www.hes-so.ch/

11. * Review team members and their organisational affiliations.

Give the personal details and the organisational affiliations of each member of the review team. Affiliation refers to groups or organisations to which review team members belong. **NOTE: email and country are now mandatory fields for each person.**

Mr David Bellagamba. University of Applied Sciences (Switzerland)
Miss Line Vionnet. University of Applied Sciences (Switzerland)
Dr Paul Vaucher. School of Health Sciences (Fribourg), University of Applied Sciences (Switzerland)

12. * Funding sources/sponsors.

Give details of the individuals, organizations, groups or other legal entities who take responsibility for initiating, managing, sponsoring and/or financing the review. Include any unique identification numbers assigned to the review by the individuals or bodies listed.

The project is funded internally by the University of Applied Sciences and Arts Western Switzerland

Grant number(s)

13. * Conflicts of interest.

List any conditions that could lead to actual or perceived undue influence on judgements concerning the main topic investigated in the review.

None

14. Collaborators.

Give the name and affiliation of any individuals or organisations who are working on the review but who are not listed as review team members. **NOTE: email and country are now mandatory fields for each person.**

Dr Nicolas Kühne. University of Applied Sciences (Switzerland)
Mr Olivier Contal. University of Applied Sciences (Switzerland)

15. * Review question.

State the question(s) to be addressed by the review, clearly and precisely. Review questions may be specific or broad. It may be appropriate to break very broad questions down into a series of related more specific questions. Questions may be framed or refined using PI(E)COS where relevant.

Primary question: What are the available and published standardized on-road instruments for assessing fitness-to-drive in people with suspected or objectified cognitive deficits following an acquired brain injury, cognitive decline or dementia?

Secondary questions:What are their psychometric properties?To what extent can they be implemented in clinical settings?

16. * Searches.

State the sources that will be searched. Give the search dates, and any restrictions (e.g. language or publication period). Do NOT enter the full search strategy (it may be provided as a link or attachment.)

The following databases will be searched: PubMed, CINAHL, PsycINFO, ISI Web of Knowledge and

Scopus/Embase.

No publication period restrictions will be used, and only studies in English or French will be included.

As the interest is in available tools, the grey literature will not be checked="checked" value="1".

Backward and forward reference searching will be used with each full text included. Forward reference searching will be done with Google Scholar.

Two main categories of keywords are used (evaluation and driving). An additional category related to the population (cognitive deficits) will be used if the equation with two categories provides too many results. The Boolean operators “AND” and “OR” will be used: “AND” between each category, and “OR” between each keyword.

If possible, a truncation will be used in order to include all potential variations of a keyword. When too many variations exist, each variation of interest will be added to the search equation.

Those keywords have been defined by testing equations on the five databases in order to find studies that were already known about this subject. New keywords have been added when these articles couldn’t be found.

Sensitivity analysis:To test the sensitivity of our search strategy, we verified if 12 known articles (Barco et al., 2015; Berndt, May & Darzins, 2015; Hunt, Morris, Edwards & Wilson, 1993; Justiss, Mann, Stav & Velozo, 2006; Kay, Bundy, Clemson & Jolly, 2008; Mallon & Wood, 2004; Odenheimer et al., 1994; Patomella & Bundy, 2015; Patomella, Tham, Johansson & Kottorp, 2010; Richardson & Marottoli, 2003; Vaucher et al., 2015; Vlahodimitrakou et al., 2013) on the topic were identified.

The search terms were improved until all studies could be identified using the search strategy.

The keywords were the following:Evaluation:record*, assess*, test, tests, testing, screen*, evaluation*, scale*, tool*, instrument*, measur*Driving:"driving error*", "on-road", driving, "route design", driver*, route, "driving performance", "open-road", "driving ability"Cognitive deficits:"cognitive impairment*", "cognitive deficit*", "mild cognitive impairment*", MCI, attention, memory, flexibility, "executive function*", cognitiv*, "cognitive concerns", "cognitive predictors", "neuropsychological deficit*" Third category of keywords (cognitive deficits) will be used only in case too many articles are identified by the database research.

17. URL to search strategy.

Give a link to a published pdf/word document detailing either the search strategy or an example of a search strategy for a specific database if available (including the keywords that will be used in the search strategies), or upload your search strategy.Do NOT provide links to your search results.

Alternatively, upload your search strategy to CRD in pdf format. Please note that by doing so you are consenting to the file being made publicly accessible.

Do not make this file publicly available until the review is complete

18. * Condition or domain being studied.

Give a short description of the disease, condition or healthcare domain being studied. This could include health and wellbeing outcomes.

Driving is an important part of the senior's community mobility. However, the prevalence of several diseases is greater in this population (e.g. dementia or strokes). These diseases can influence the driving performance. The crash risk is then increased and thus reduce road safety. For this reason, it is essential to assess senior's driving performance. On-road evaluation has been considered for several years as the gold standard. Many on-road instruments have been developed recently.As population ages in Switzerland and as the rate of seniors having a driving license increase, it is important to set up a standardised on-road evaluation. There is at the moment no on-road assessment to support decision making concerning driving cessation. The aim of this systematic review is to identify those instruments and make recommendations in order to set up an on-road evaluation that is usable by occupational therapists in French-speaking Switzerland.

19. * Participants/population.

Give summary criteria for the participants or populations being studied by the review. The preferred format includes details of both inclusion and exclusion criteria.

People with cognitive deficits, resulting for example from a stroke, a traumatic brain injury or a dementia, or based on the assessments of an expert committee. No restrictions will be imposed on age or gender.

20. * Intervention(s), exposure(s).

Give full and clear descriptions or definitions of the nature of the interventions or the exposures to be reviewed.

Standardized assessment tools of on-road driving performance.

21. * Comparator(s)/control.

Where relevant, give details of the alternatives against which the main subject/topic of the review will be compared (e.g. another intervention or a non-exposed control group). The preferred format includes details of both inclusion and exclusion criteria.

Not applicable.

22. * Types of study to be included.

Give details of the types of study (study designs) eligible for inclusion in the review. If there are no restrictions on the types of study design eligible for inclusion, or certain study types are excluded, this should be stated. The preferred format includes details of both inclusion and exclusion criteria.

Inclusion

(1) Studies involving any validation of an on-road fitness-to-drive assessment tool;

(2) Studies which include assessment tools which are suitable for use with people with cognitive deficits;

(3) Studies written in English or French.

No publication period restrictions will be imposed.

Exclusion:

(1) Studies involving simulator assessments;

(2) Studies involving licensing evaluations;

(3) Studies involving a specific topic, such as drug use with respect to fitness-to-drive.

23. Context.

Give summary details of the setting and other relevant characteristics which help define the inclusion or exclusion criteria.

24. * Main outcome(s).

Give the pre-specified main (most important) outcomes of the review, including details of how the outcome is defined and measured and when these measurement are made, if these are part of the review inclusion criteria.

This study is a clinimetric review and reports psychometric values of existing instruments to evaluate on-road driving performance. As such, we are mainly interested in extracting summary measures for consistency, coherence, reliability and validity.

Clinimetric reviews aim to identify and extract data on validity of clinical instruments (ex: PMID 28490031).

To our knowledge, there is no clear existing guidelines for clinimetric reviews (Cochrane article on the subject : https://abstracts.cochrane.org/2006-dublin/assessment-methodological-quality-clinimetric-reviews-systematic-review). The closest we have come to providing guidelines is the Cochrane Handbook for Systematic Reviews of Diagnostic Test Accuracy [1]. There are however no indication towards defining primary outcomes. Furthermore, clinimetrics is also applicable to measure phenomenons that are not a diagnosis. In this clinimetric review, we intend to extract information on the validity of measuring instrument evaluating on-road driving performance.

Reference:

Reitsma JB, Rutjes AWS, Whiting P, Vlassov VV, Leeflang MMG, Deeks JJ,. Chapter 9: Assessing methodological quality. In: Deeks JJ, Bossuyt PM, Gatsonis C (editors), Cochrane Handbook for Systematic Reviews of Diagnostic Test Accuracy Version 1.0.0. The Cochrane Collaboration, 2009. Available from: http://srdta.cochrane.org/.

* Measures of effect

Please specify the effect measure(s) for you main outcome(s) e.g. relative risks, odds ratios, risk difference, and/or 'number needed to treat.

Not applicable.

25. * Additional outcome(s).

List the pre-specified additional outcomes of the review, with a similar level of detail to that required for main outcomes. Where there are no additional outcomes please state ‘None’ or ‘Not applicable’ as appropriate to the review

None.

* Measures of effect

Please specify the effect measure(s) for you additional outcome(s) e.g. relative risks, odds ratios, risk difference, and/or 'number needed to treat.

Not applicable.

26. * Data extraction (selection and coding).

Describe how studies will be selected for inclusion. State what data will be extracted or obtained. State how this will be done and recorded.

The references retrieved during the searches will be screened first by titles, and then by abstracts, to identify potentially relevant articles. The full texts of studies deemed to be possibly eligible for inclusion will then be obtained and assessed for inclusion/exclusion.

Additional studies will be selected by screening the references of the studies evaluated as full texts. In order to ensure that the psychometric properties of each assessment are recorded comprehensively, validation studies will then be selected for each one identified.

Studies will be hand-searched separately by the two main authors, and, in case of any discrepancies arising, consensus will be reached by discussion/debate. If consensus cannot be reached between the two authors, the point of view of the thesis director (PV) will be requested.

The data to be extracted will be as follows:

General information:- Name of the test;- Authors and year of publication;- Sample;- Context;- Available versions.

Road test:- Type of evaluation (open or closed);- Route design;- Distance;- Duration;- Description of the procedure;- Diversity, difficulty, adaptability.

Assessment tool:- Number of items;- Score ;- Cut-off score;- Modifications (if several articles).

Psychometric data:- Reliability;- Validity.

Implementability:- Acceptability;- Accessibility;- Cost;- Prerequisites/training.

27. * Risk of bias (quality) assessment.

Describe the method of assessing risk of bias or quality assessment. State which characteristics of the studies will be assessed and any formal risk of bias tools that will be used.

Study selection and appraisal, and data extraction will be conducted by two researchers independently, and in duplicate by members of the research team. Discrepancies will be addressed by consulting the thesis director (PV). The COSMIN four-point rating scale will be used to assess the quality of the included studies. Developed in 2011, this scale provides a global score for each assessment tool and thus provides an overall methodological quality score. None of the authors of this systematic review have been involved in the development of the included assessment tools.

28. * Strategy for data synthesis.

Provide details of the planned synthesis including a rationale for the methods selected. This **must not be generic text** but should be **specific to your review** and describe how the proposed analysis will be applied to your data.

We do not expect more than two to three studies to have evaluated similar psychometrics for the same instrument, and as such, we will use a narrative descriptive synthesis of aggregated participant data. The different assessment tools will be compared by considering the data extracted. Furthermore, expert recommendations from the available literature will be used to ensure that the included assessment tools meet the essential criteria. The COSMIN results will also be compared in order to recommend an assessment with sound psychometric properties, and implementability criteria will also be taken into account.

29. * Analysis of subgroups or subsets.

State any planned investigation of ‘subgroups’. Be clear and specific about which type of study or participant will be included in each group or covariate investigated. State the planned analytic approach.

None planned.

30. * Type and method of review.

Select the type of review and the review method from the lists below. Select the health area(s) of interest for your review.

Type of review

Cost effectiveness
No

Diagnostic
Yes

Epidemiologic
No

Individual patient data (IPD) meta-analysis
No

Intervention
No

Meta-analysis
No

Methodology
No

Narrative synthesis
Yes

Network meta-analysis
No

Pre-clinical
No

Prevention
No

Prognostic
No

Prospective meta-analysis (PMA)
No

Review of reviews
No

Service delivery
No

Synthesis of qualitative studies
No

Systematic review
Yes

Other
No

Health area of the review

Alcohol/substance misuse/abuse
No

Blood and immune system
No

Cancer
No

Cardiovascular
No

Care of the elderly
No

Child health
No

Complementary therapies
No

Crime and justice
No

Dental
No

Digestive system
No

Ear, nose and throat
No

Education
No

Endocrine and metabolic disorders
No

Eye disorders
No

General interest
No

Genetics
No

Health inequalities/health equity
No

Infections and infestations
No

International development
No

Mental health and behavioural conditions
No

Musculoskeletal
No

Neurological
Yes

Nursing
No

Obstetrics and gynaecology
No

Oral health
No

Palliative care
No

Perioperative care
No

Physiotherapy
No

Pregnancy and childbirth
No

Public health (including social determinants of health)
No

Rehabilitation
Yes

Respiratory disorders
No

Service delivery
No

Skin disorders
No

Social care
No

Surgery
No

Tropical Medicine
No

Urological
No

Wounds, injuries and accidents
No

Violence and abuse
No

31. Language.

Select each language individually to add it to the list below, use the bin icon to remove any added in error.
English
French

There is not an English language summary

32. * Country.

Select the country in which the review is being carried out from the drop down list. For multi-national

collaborations select all the countries involved.

Switzerland

33. Other registration details.

Give the name of any organisation where the systematic review title or protocol is registered (such as with The Campbell Collaboration, or The Joanna Briggs Institute) together with any unique identification number assigned. (N.B. Registration details for Cochrane protocols will be automatically entered). If extracted data will be stored and made available through a repository such as the Systematic Review Data Repository (SRDR), details and a link should be included here. If none, leave blank.

34. Reference and/or URL for published protocol.

Give the citation and link for the published protocol, if there is one

Give the link to the published protocol.

Alternatively, upload your published protocol to CRD in pdf format. Please note that by doing so you are consenting to the file being made publicly accessible.

No I do not make this file publicly available until the review is complete

Please note that the information required in the PROSPERO registration form must be completed in full even if access to a protocol is given.

35. Dissemination plans.

Give brief details of plans for communicating essential messages from the review to the appropriate audiences.

The results of this systematic review will be presented during a thesis defence in spring 2019 in Lausanne, Switzerland. If the quality of the thesis is good enough, a public open access publication will also be considered.

Do you intend to publish the review on completion?

Yes

36. Keywords.

Give words or phrases that best describe the review. Separate keywords with a semicolon or new line. Keywords will help users find the review in the Register (the words do not appear in the public record but are included in searches). Be as specific and precise as possible. Avoid acronyms and abbreviations unless these are in wide use.

Systematic review; Driving assessment; Evaluation; Fitness-to-drive; On-road; Psychometric properties

37. Details of any existing review of the same topic by the same authors.

Give details of earlier versions of the systematic review if an update of an existing review is being registered, including full bibliographic reference if possible.

38. * Current review status.

Review status should be updated when the review is completed and when it is published. For newregistrations the review must be Ongoing.
Please provide anticipated publication date

Review_Completed_not_published

39. Any additional information.

Provide any other information the review team feel is relevant to the registration of the review.

This review is being undertaken in order to consider an assessment to implement in the French-speaking Switzerland.

Review team members:

Master's degree candidates:

Mr David Bellagamba (named contact):

Email: bellagamba.david@gmail.com.

Miss Line Vionet:

Email: line.vionnet@gmail.com;

Telephone: +41 79 221 68 53 or +41 78 845 34 59.

Master's thesis director:

Dr Paul Vaucher:

Email: paul.vaucher@hes-so.ch;

Telephone: +41 78 788 33 66.

40. Details of final report/publication(s).

This field should be left empty until details of the completed review are available.

Give the link to the published review.
